# An Investigation of Differences in Lower Extremity Biomechanics During Single-Leg Landing From Height Using Bionic Shoes and Normal Shoes

**DOI:** 10.3389/fbioe.2021.679123

**Published:** 2021-08-09

**Authors:** Datao Xu, Huiyu Zhou, Julien S. Baker, Bíró István, Yaodong Gu

**Affiliations:** ^1^ Faculty of Sports Science, Ningbo University, Ningbo, China; ^2^ School of Health and Life Sciences, University of the West of Scotland, Scotland, United Kingdom; ^3^ Centre for Health and Exercise Science Research, Department of Sport and Physical Education, Hong Kong Baptist University, Hong Kong, China; ^4^ Faculty of Engineering, University of Szeged, Szeged, Hungary

**Keywords:** unstable sole construction, bionic shoes, landing task, sports injury, lower limb

## Abstract

Bionic shoes utilizing an actual foot shape sole structure can alter lower limb’s biomechanics, which may help in the development of specific training or rehabilitation programs. The purpose of this study was to investigate the biomechanical differences in the lower limb during a single-leg landing task using bionic shoes (BS) and normal shoes (NS). Fifteen healthy male subjects participated in this study, sagittal, and frontal plane data were collected during the landing phase (drop landing from 35 cm platform). Our study showed that BS depicted a significantly greater minimum knee flexion angle at initial contact (*p* = 0.000), a significantly greater minimum (initial contact) hip flexion angle at initial contact (*p* = 0.009), a significantly smaller sagittal plane total energy dissipation (*p* = 0.028), a significantly smaller frontal plane total energy dissipation (*p* = 0.008), a significantly smaller lower limb total energy dissipation (*p* = 0.017) than NS during the landing phase. SPM analysis revealed that BS depicted a significantly smaller knee joint vertical reaction force during the 13.8–19.8% landing phase (*p* = 0.01), a significantly smaller anterior tibia shear force during the 14.2–17.5% landing phase (*p* = 0.024) than NS. BS appears to change lower limb kinematics at initial contact and then readjust the landing strategies for joint work and joint reaction force, thereby reducing the risk of lower limb skeletal muscle injury. BS have great potential for future development and application uses, which may help athletes to reduce lower limb injury risk.

## Introduction

Landing is essential in a variety of sports, including landing with single-leg and landing with double-legs. However, the landing process is often accompanied by a high rate and high-intensity musculoskeletal load impact, which often causes a large degree of muscle damage to the musculoskeletal system (Zhang et al., 2000; Shimokochi et al., 2013), such as ligament damage, achilles tendon inflammation and joint pain (Radin et al., 1984; Dufek and Bates, 1991; Radin et al., 1991). These different injury patterns are the result of transmission development in impact load on the lower extremities. This is related to the fact that the kinetic energy of downward acceleration is dissipated by a combination of myofibrillar contraction and skeletal muscle structure during landing (Zhang et al., 2000). When landing on a single-leg, this is accompanied by a higher load because the limb can only absorb the impact on the side being used. At the same time, landing with a single-leg will often cause the lower limb joints to be unstable and bear greater impact loads, thereby increasing the risk of lower limb injury (Yeow et al., 2011; Xu et al., 2021b).

In particular, the knee joint is a joint capsule with multiple joints, which is easily damaged during the energy impact and transmission of the lower limb kinematic chain (Guskiewicz and Perrin, 1996; Riemann and Lephart, 2002; Baker et al., 2021). Many researchers focused on knee anterior cruciate ligament (ACL) injuries for many years, especially the ACL injury caused by the higher joint reaction force. Landing from height is one of the most important risk factors for ACL injury. In many common sports, such as basketball, volleyball, and football et al., athletes have suffered ACL injuries due to landing, and 70% have occurred under non-contact conditions. Another reason why ACL injuries are of great concern to researchers is the increased risk of secondary injuries. Previous studies have followed up patients who accepted ACL surgery reconstruction (Paterno et al., 2014; Webster and Feller, 2016). They found a 30–35% chance of the patient suffering second ACL injuries. Most researchers have been interested in how to reduce ACL injuries and this is a major problem area to prolong athletic careers (Devita and Skelly, 1992; Chappell et al., 2002).

A successful landing task requires sufficient muscle strength and joint stability to prevent lower limb injuries and exhibits greater knee and hip flexion to cushion the load (Wikstrom et al., 2004). During the landing process, the lower extremities bear the distal load to the proximal end, first from the foot to the ankle, then the knee, and finally to the hip. Therefore, the interaction between the foot and the ground plays an essential role in the musculoskeletal system load (Powell et al., 2012). The importance of shoes as a medium connecting the feet and the ground is self-evident. As an essential item in daily life, a shoe designs fundamental role is to protect the feet and maintain posture and stability (Reinsch and Nigg, 2000). With the expansion of various sports equipment demands, different functional sports shoes (such as basketball shoes, football shoes, running shoes, etc.) have been developed. Shoes for daily walking and various sports are designed to provide comfort and stability using flat and different hardness soles (Romkes et al., 2006). Therefore, some researchers have proposed using shoes with special design concepts to reduce landing injuries.

Normal footwear provides the functions of protection and supports the foot, which could lead to overprotection and restraint of the foot. This condition may lead to decreased function of the lower limb muscles, reduced muscle strength, and increased risk of potential musculoskeletal injury. (Nigg et al., 2006a; Sousa and Tavares, 2012). Based on these concerns, a growing number of studies have focused on the effects of unstable shoes, such as Masai Barefoot Technology (MBT) and Reflex Control Shoes (RC), which play an important role in injury prevention and balance control in dynamic postures (Decker et al., 2002; Turbanski et al., 2011). Previous studies have shown that unstable shoes can increase muscle activity *via* co-contraction between the antagonist and agonist at each joint (Horsak et al., 2015). The increased muscle activity may alter landing strategies to reduce landing injuries and thus serve as a potential protective mechanism. A growing body of research has shown that unstable shoes can produce definite instability, which effectively improves proprioception, then increases postural stability and reduces the perceived level of pain. It also improves muscle coordination in both the lower extremities and the body generally (Stöggl et al., 2010; Turbanski et al., 2011; Li et al., 2015; Mei et al., 2015; Zhang et al., 2017). The impact of all these changes on the lower extremities is undoubtedly significant, but few studies have investigated the impact of unstable shoes on landing.

The barefoot shoe was one of the first ideas to create unstable shoes. Over the years, it is necessary to protect the foot because the cuticle of the human foot becomes degraded. Simultaneously, the idea of an unstable shoe is also derived from an unstable training device, whose sole structure alters the lower limb’s biomechanics and may help in the development of specific training or rehabilitation programs (Gu et al., 2014). The soles of the feet play an important role in providing valuable tactile feedback to the central nervous system (Zhang and Li, 2013). Bionics is an interdisciplinary subject that combines engineering and biological sciences, which has been widely developed in recent years because of design specificity concerning certain products according to the needs of users. Therefore, we proposed a new bionic design scheme based on unstable shoes and barefoot shoes. We call this personalized design of the foot’s structure and shape on the sole a bionic shoe. Compared to the general unstable shoes, the bionic shoes with a personalized bionic customized signature are a structure that both combines the barefoot design of barefoot shoes and the unstable structure of unstable shoes. Zhou further explains that the most significant difference between unstable and bionic shoes is that bionic shoes combined the benefit of original human walking and unstable shoes (Zhou et al., 2021a). This kind of bionic shoe adopts the personalized foot shape as the outsole can restore the original barefoot condition of humans to the greatest extent and may reduce injury in the human lower limbs. Therefore, bionic shoes have been widely developed, researched, and promoted for their comprehensive advantages, such as alleviating muscle deterioration.

Previous studies on biomechanical differences that bionic shoes may cause have been conducted, but most of them have focused on walking and running (Zhou et al., 2018; Jiang et al., 2021), and experimental evidence is lacking concerning landing from heights. The interaction between the foot and the ground as the initial contact point plays a crucial role in this energy transfer process (Powell et al., 2012). It determines the energy dissipation strategy of lower extremity joints and lower extremity joints’ influence on shock absorption during landing (Lee et al., 2018). The lower limb joint energy dissipation and shock absorption mainly manifest in the sagittal and frontal planes during landing (Yeow et al., 2011). Previous studies have also shown that lower limb joint reaction force is a critical index to evaluate lower limb injury risk. The higher joint reaction force is considered an important characteristic that contributes to increased injury risk (Chappell et al., 2002; Mei et al., 2019). Therefore, it is essential to understand if bionic shoes can adjust lower limb joints’ landing strategies, such as the effects on the sagittal and frontal planes’ shock absorption dynamics during landing and changes in the joint reaction force during landing tasks. This may help develop sports equipment and reduce lower limb injury in athletes.

Therefore, given the lack of knowledge about landing from height using bionic shoes, the purpose of this study was to investigate the effects of bionic shoes on the biomechanics of the lower limb sagittal plane and frontal plane during landing. Compared with normal shoes (NS), bionic shoes (BS) are completely consistent with normal shoes in all other aspects except that the sole is designed according to personalized foot structure and shape. We hypothesized that the lower limb sagittal and frontal joint angle, joint moment, joint power, joint work values, joint reaction force, and contributions to load attenuation using NS and BS might be different. The differences may also reduce landing injury from the perspective of biomechanics. Therefore, given the lack of knowledge about landing from height using bionic shoes, the purpose of this study was to investigate the effects of bionic shoes on the biomechanics of the lower limb sagittal plane and frontal plane during landing. Compared with normal shoes (NS), the bionic shoes (BS) are completely consistent with normal shoes in all other aspects except that the sole is designed according to personalized foot structure and shape. We hypothesized that the lower limb sagittal and frontal joint angle, joint moment, joint power, joint work values, joint reaction force, and contributions to load attenuation using NS and BS might be different. The differences may also reduce landing injury from the perspective of biomechanics.

## Materials and Methods

### Participants

A total of 15 healthy male subjects participated in this study (Age: 23.1 ± 1.7 years, height: 1.78 ± 0.05 m, body mass: 74.2 ± 7.5 kg). All subjects were amateur athletes (performed a moderate or above physical strength exercise for at least 30 min at least three times a week). There was no history of lower extremity surgery. No lower extremity injuries or pain were reported in the previous 6 months. No medical problems (e.g., osteoarthritis, diabetes, and neurological disorders) that might affect their performance were reported. Prior to the experiment, all subjects were required to run or jump with bionic shoes at least 1 h per day for 2 weeks to allow for adaptation to the shoes. All subjects were informed of the purpose, requirements, and procedures of the study. All subjects provided written informed consent and the Ethics Committee at Ningbo University approved this study.

### Testing Shoes

**FIGURE 1:**
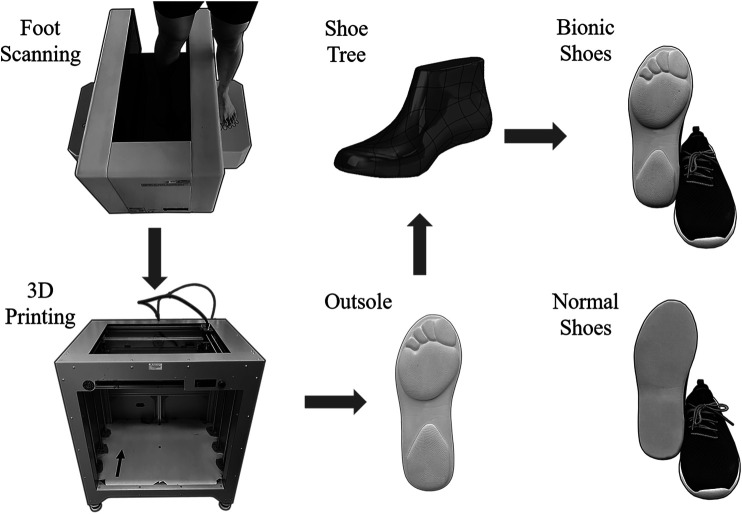
Illustration of BS and NS making procedure from initial idea to finished product.

Our study’s testing shoes included two different types of shoe: Normal shoes (NS) and Bionic Shoes (BS). The BS were customized for each participant according to their foot structure and shape. Figure 1 shows the procedure for the development of BS and NS. The process of making shoes mainly includes the following steps: 1) Firstly, a foot scanning machine (VAS-39, Ortho baltic, LITHUANIA) was used to scan individual foot structure and shape; 2) Secondly, 3D print [Dragon(L) 3D Printer, WINBO, China] was used to print the plastic foot model based on the data from the foot scanner; 3) Finally, the scanned data was given to the shoe factory (Ningbo Jiangbei Feibu Sports Goods Co., Ltd. CHINA) who developed the shoe tree and then manufactured the shoe. These shoes were also produced by Ningbo Jiangbei Feibu Sports Goods Co., Ltd. (CHINA) for the NS. Compared with NS, the BS is completely consistent with normal shoes in all other aspects (e.g., materials, thickness, and hardness) except for the outsole’s shape.

### Experimental Protocol and Procedures

**FIGURE 2:**
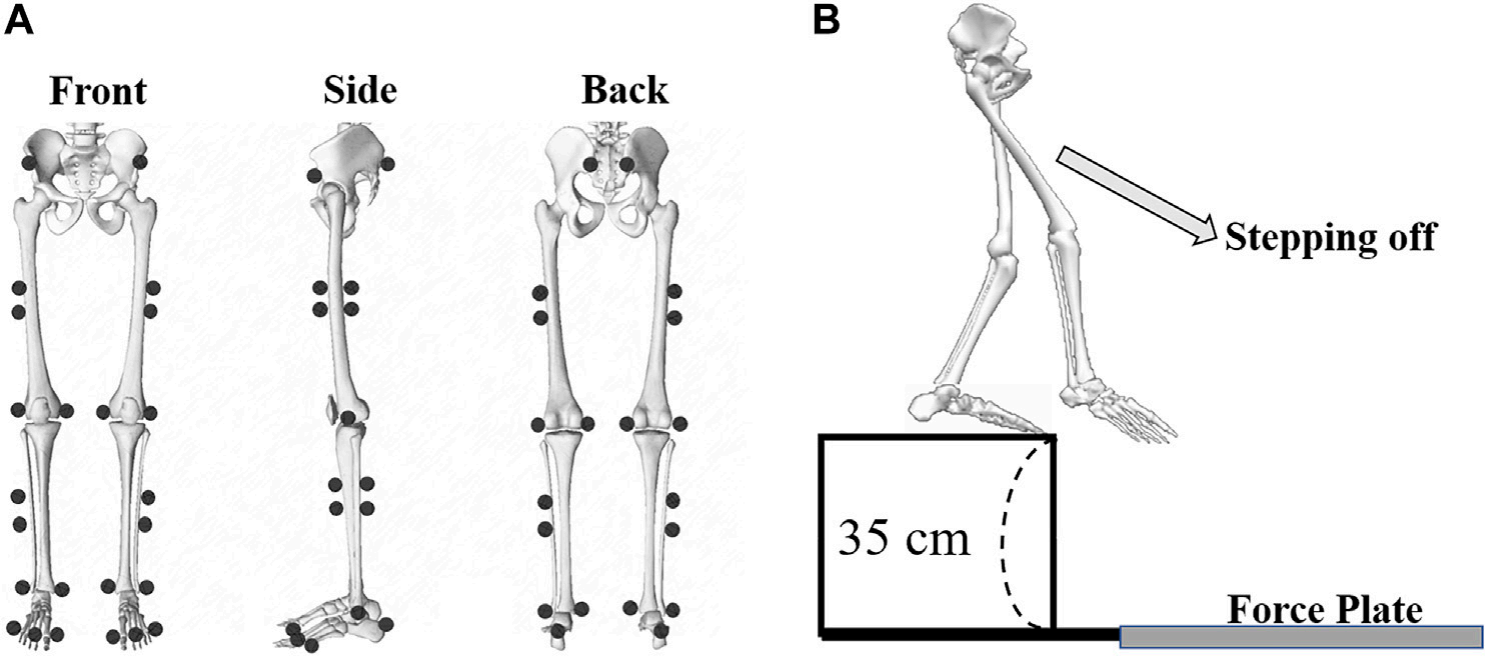
**(A)**: The reflective markers placement (front view, side view, and back view) following bony anatomical landmarks; **(B)**: The process of subjects conducting single-leg drop landing maneuvers by stepping off a 35 cm platform and landing with the dominant leg onto the center of the force plate.

The experiment was carried out in the biomechanics laboratory at Ningbo University. The height and body mass of subjects were measured with a stadiometer and calibrated scale. Based on a previous study (Xu et al., 2021a), a total of thirty-six standard reflective markers (diameter: 12.5 mm) were labeled to the bilateral lower limbs and pelvis to track the lower limb’s motion trajectory. Figure 2A shows the reflective marker placement. The reflective marker locations included right and left anterior superior iliac spine and posterior superior iliac spine, right and left medial and lateral condyle, right and left medial and lateral malleolus, first and fifth metatarsal heads, distal interphalangeal joint of the second toe. Six Tracking clusters were placed on the right and left middle and lateral thigh, shank, and heel. An eight Infrared camera Vicon motion capture system (Vicon Metrics Ltd., Oxford, United Kingdom) was used to capture the trajectory of motion, and kinetic data was obtained using an in-ground force plate (AMTI, Watertown, MA, United States). Vicon Nexus 1.8.6 software was used to collect kinematics and kinetic data synchronously, and the sampling frequency of kinematics and kinetic was established at 200 and 1000Hz, respectively.

Before the formal experiment, participants were required to wear uniform tights and leggings, to warm up for 10 min at a speed of 8 km/h on a treadmill, and then perform full muscle stretching. Then, all participants familiarized themselves with the experimental environment and practiced a drop landing task until they were familiar with test movements. According to previous studies, most subjects selected 30–40 cm as drop landing heights (Mason et al., 2017; Collings et al., 2019). We also consider that this height can effectively detect the differences between bionic shoes and minimize the damage to subjects caused by repeated landing experiments. Too low a height cannot effectively detect any performance differences, while too high a height will cause inevitable damage to the subjects, so we chose the height of 35 cm. Therefore, A platform (height: 35 cm) was placed in front of the force plate, located within the center of the eight infrared cameras. Figure 2B shows the process of the landing task. The participants were instructed to stand on the platform and then perform a drop landing protocol using a single-leg by stepping off of the platform. The dominant leg (the preferred leg in a daily exercise that is better suited for landing or kicking a ball) of the subjects was used to land on the force plate. In order to reduce possible errors, the landing sequence with NS and BS was randomly assigned. A successful trial was defined when the participants stepped off the platform with arms crossed in front of their chest and then maintained balance on their dominant leg for 3 s after landing. Subjects were allowed a 30 s rest between each landing protocols, and five successful landing data sets were obtained for each participant using BS or NS. A total of ten data sets were collected.

### Data Collection and Processing

According to a previous study, we defined that the vertical ground reaction force (VGRF) exceeding 10 N as the initial contact point (Xu et al., 2020). The landing phase was defined as the initial contact point to maximum knee flexion. A total of 5 s of data was collected, including 2 s before the initial contact with the ground and 3 s after the contact with the ground. The data for the movement of reflective markers and the ground reaction force were processed using Vicon Nexus 1.8.6 software, and the C3D format file was exported after processing. The C3D file was imported into Visual 3-D software (version 4.96, C-Motion Inc., Germantown, MD, United States) for static modeling and further processing. Based on Winter’s description of the selected frequency of the filter (Winter, 2009), the residual analysis of data was carried out in subsets to determine the most appropriate signal-to-noise ratio. The kinematics and kinetic data were filtered using 10 and 20 Hz fourth-order zero-phase lag Butterworth low-pass filters.

The CODA pelvis defined the location of the hip joint centers using regression equations according to Bell and Brand’s previous research (Bell et al., 1989; Bell et al., 1990). Estimates for the right hip joint center (RHJC) and left hip joint center (LHJC) were defined by the anterior superior iliac spine (ASIS). As shown in Eq. 1 (RHJC) and Eq. 2 (LHJC):

$$\mathrm{RHJC}=\left(0.36*\mathrm{ASI}S_{\mathrm{Distance}},\;-0.19*\mathrm{ASI}S_{\mathrm{Distance}},\;-0.3*\mathrm{ASIS}\_\mathrm{Distance}\right)$$ (1)

$$\mathrm{LHJC}=\left(-0.36*\mathrm{ASI}S_{\mathrm{Distance}},\;-0.19*\mathrm{ASI}S_{\mathrm{Distance}},\;-0.3*\mathrm{ASIS}\_\mathrm{Distance}\right)$$ (2)

The markers’ location determined the joint centers of the other joints, and the length of the bone was determined from the joint centers of each joint. The joint angle, joint reaction force, joint moment, and joint power were calculated using an inverse kinetics algorithm, which was conducted using Visual 3-D software. Finally, all data exported from V3D were then imported into MATLAB R2019a (MathWorks, MA, United States) to process further. The code of MATLAB processing method was our custom script, and the whole data processing process used MATLAB scripts to reduce the errors caused by manual processing. In addition, a custom MATLAB script was used to intercept the time point between the initial contact with the ground and the knee joint’s maximum flexion position during the landing process.

Positive values were defined as knee and hip extension and ankle plantarflexion for the sagittal plane. The negative value was defined as knee and hip flexion and ankle dorsiflexion. For the frontal plane, the positive value was defined as knee and hip abduction and ankle eversion. The negative value was defined as knee and hip adduction and ankle inversion. The integral of joint power over time and joint work was calculated, and negative (eccentric) work reflected the joint muscles’ energy dissipation (Xu et al., 2021b). The individual joint work contribution to the total energy dissipation was calculated as the percentage of the joint energy dissipation in the total energy dissipation of the ankle, knee, and hip joints. The negative work values indicated energy dissipation through eccentric muscular contraction (Winter, 2009). Joint work was normalized to body mass (BM). The knee joint reaction force calculated by inverse dynamics was translated to the tibial reference frames and deconstructed into anterior tibial shear force (ATSF) (Chappell et al., 2002; Zhou et al., 2021b). All the data interpretations were calculated using MATLAB scripts.

### Statistical Analysis

To ensure that the data was normally distributed prior to statistical analysis, all data (peak joint angle, peak joint moment, peak joint power, mean energy dissipation value, and contribution ratio to total energy dissipation) were verified using the Shapiro-Wilk normality test. For non-parametric data, the Wilcoxon matched-pairs signed-rank test was conducted.

For traditional analysis (peak variables), a paired-samples *t*-test was used to (SPSSs Inc., Chicago, IL, United States) analyze all data. The threshold of significance was set at 0.05 (*α* = 0.05). The data for the comparative analysis in this study were collected based on actual observations of the experiment, rather than random numbers, which resulted in fewer interpretation errors. Therefore, the data analysis in this study was not adjusted by multiple comparisons and corrections (Rothman, 1990; Perneger, 1998). For statistical parametric mapping of one-dimensional (SPM 1 day) analysis, all curves of the time series were extracted to expand into 101 data points (represent the 0–100% landing phase) using a custom MATLAB script. Then, the open-source MATLAB script of SPM 1 day paired-samples *t*-test was used in the statistical analysis (significance threshold set as 0.05) (Pataky, 2010; Pataky et al., 2013).

## Results

### Joint Angles, Joint Moment, and Joint Power

For the sagittal joint angle, Table 1 shows that BS depicted a significantly smaller peak dorsiflexion angle (*p* = 0.049), a significantly greater initial contact knee flexion angle (*p* = 0.000), a significantly greater initial contact hip flexion angle (*p* = 0.009), a significantly greater peak hip flexion angle (*p* = 0.005) than NS during the landing phase. For the sagittal plane peak joint moment and power, Table 1 shows that BS depicted a significantly smaller peak ankle joint power (*p* = 0.005), a significantly smaller peak knee joint moment (*p* = 0.001), a significantly smaller peak knee joint power (*p* = 0.042), a significantly smaller peak hip joint power (*p* = 0.019) than NS during the landing phase. There were no differences for the peak ankle moment and peak hip joint moment.

**TABLE 1:** Comparison of sagittal plane joint angles, joint moment, joint power (means ± standard) between NS and BS during single-leg landing phase.

Biomechanical variables		Normal shoe	Bionic shoe	Difference value	P
Ankle joint	Peak plantarflexion angle (°)	31.75 ± 4.71	32.73 ± 5.34	0.98	0.311
	Peak dorsiflexion angle (°)	−26.52 ± 2.36	−24.66 ± 3.01	−1.86	0.049^a^
	Range of joint motion (°)	58.27 ± 5.27	57.39 ± 4.93	−1.33	0.400
	Peak joint moment (Nm/kg)	1.24 ± 0.34	1.20 ± 0.22	−0.04	0.064
	Peak joint power (W/kg)	−8.54 ± 2.31	−5.53 ± 1.07	−3.01	0.005^a^
Knee joint	Initial contact flexion angle (°)	−9.35 ± 3.63	−13.74 ± 4.17	4.39	0.000^a^
	Peak flexion angle (°)	−78.95 ± 6.07	−81.21 ± 7.11	2.26	0.133
	Range of joint motion (°)	69.61 ± 7.30	68.46 ± 8.46	−1.15	0.360
	Peak joint moment (Nm/kg)	2.58 ± 0.49	2.26 ± 0.32	−0.32	0.001^a^
	Peak joint power (W/kg)	−21.33 ± 3.69	−17.34 ± 3.51	−3.99	0.042^a^
Hip joint	Initial contact flexion angle (°)	−13.99 ± 3.93	−16.81 ± 3.37	2.82	0.009^a^
	Peak flexion angle (°)	−48.48 ± 4.92	−53.63 ± 5.25	5.15	0.005^a^
	Range of joint motion (°)	37.49 ± 5.62	36.82 ± 6.02	−0.67	0.879
	Peak joint moment (Nm/kg)	2.13 ± 0.58	2.22 ± 0.69	0.09	0.849
	Peak joint power (W/kg)	−17.10 ± 3.25	−13.22 ± 2.55	−3.88	0.019^a^

Note: °: Degrees; Nm/kg: N m per kilogram; W/kg: Watts per kilogram. Difference value: The absolute value of the bionic shoe minus the absolute value of the normal shoe.

a means significance with *p* < 0.05.

For the frontal joint angle, Table 2 shows that BS depicted a significantly smaller peak eversion angle (*p* = 0.000), a significantly greater peak inversion angle (*p* = 0.034) than NS during the landing phase. For the frontal plane the peak joint moment and power, Table 2 shows that BS depicted a significantly smaller peak knee joint moment (*p* = 0.000), a significantly smaller peak knee joint power (*p* = 0.022), a significantly smaller peak hip joint moment (*p* = 0.062), a significantly smaller peak hip joint power (*p* = 0.000) than NS during the landing phase.

**TABLE 2:** Comparison of frontal plane joint angle, joint moment, joint power (means ± standard) between NS and BS during single-leg landing phase.

Biomechanical variables		Normal shoe	Bionic shoe	Difference value	P
Ankle joint	Peak eversion angle (°)	12.17 ± 3.36	6.65 ± 2.00	−5.52	0.000^a^
	Peak inversion angle (°)	−12.56 ± 4.46	−17.42 ± 5.09	4.86	0.034^a^
	Range of joint motion (°)	25.63 ± 4.27	24.08 ± 5.53	−1.55	0.41
	Peak joint moment (Nm/kg)	0.34 ± 0.08	0.33 ± 0.06	−0.01	0.537
	Peak joint power (W/kg)	−1.37 ± 0.43	−1.72 ± 0.39	0.35	0.152
Knee joint	Minimum abduction angle (°)	5.72 ± 1.34	4.90 ± 0.91	−0.82	0.130
	Maximum abduction angle (°)	14.61 ± 3.27	13.12 ± 2.50	−1.49	0.156
	Range of joint motion (°)	8.89 ± 3.67	8.22 ± 3.31	−0.67	0.550
	Peak joint moment (Nm/kg)	0.59 ± 0.19	0.30 ± 0.12	−0.29	0.000^a^
	Peak joint power (W/kg)	−5.96 ± 1.70	−4.24 ± 0.82	−1.72	0.022^a^
Hip joint	Peak abduction angle (°)	3.55 ± 1.70	3.32 ± 1.74	−0.23	0.785
	Peak adduction angle (°)	−11.50 ± 2.41	−12.82 ± 1.97	1.32	0.239
	Range of joint motion (°)	15.05 ± 3.63	16.15 ± 3.17	1.10	0.515
	Peak joint moment (Nm/kg)	1.39 ± 0.26	1.22 ± 0.33	−0.17	0.062
	Peak joint power (W/kg)	−10.92 ± 3.88	−6.36 ± 2.23	−4.56	0.000^a^

Note: °: Degrees; Nm/kg: N m per kilogram; W/kg: Watts per kilogram. Difference value: The absolute value of the bionic shoe minus the absolute value of the normal shoe.

a means significance with *p* < 0.05.

### Joint Energy Work

The results of mean energy dissipation and contribution to total energy dissipation in the sagittal plane, frontal plane and lower extremity (lower extremity energy dissipation defined as the sum of the sagittal plane and the frontal plane energy dissipation) are shown in Table 3.

**TABLE 3:** Comparison of mean energy dissipation and contribution to total energy dissipation in the sagittal plane, frontal plane, and lower extremity (means ± standard) between NS and BS during single-leg landing phase.

Biomechanical variables			Normal shoe	Bionic shoe	Difference value	P
Sagittal plane	Ankle joint	Mean energy dissipation (J/kg)	−0.77 ± 0.29	−0.61 ± 0.20	−0.16	0.119
		Contribution to total Energy dissipation (%)	27.91 ± 5.53	25.74 ± 5.34	−2.17	0.439
	Knee joint	Mean energy dissipation (J/kg)	−1.41 ± 0.38	−1.26 ± 0.32	−0.15	0.137
		Contribution to total energy dissipation (%)	51.23 ± 5.16	52.27 ± 7.27	1.04	0.748
	Hip joint	Mean energy dissipation (J/kg)	−0.57 ± 0.15	−0.49 ± 0.10	−0.08	0.245
		Contribution to total energy dissipation (%)	20.86 ± 4.40	21.99 ± 6.45	1.13	0.688
		Total energy dissipation (J/kg)	−2.75 ± 0.58	−2.31 ± 0.33	−0.44	0.028^a^
Frontal plane	Ankle joint	Mean energy dissipation (J/kg)	−0.10 ± 0.03	−0.13 ± 0.05	0.03	0.201
		Contribution to total Energy dissipation (%)	17.56 ± 3.22	29.12 ± 8.66	11.56	0.006^a^
	Knee joint	Mean energy dissipation (J/kg)	−0.20 ± 0.06	−0.15 ± 0.03	−0.05	0.044^a^
		Contribution to total energy dissipation (%)	36.22 ± 8.41	35.40 ± 7.44	−0.82	0.840
	Hip joint	Mean energy dissipation (J/kg)	−0.26 ± 0.05	−0.15 ± 0.03	−0.11	0.000^a^
		Contribution to total energy dissipation (%)	46.23 ± 7.35	35.48 ± 8.09	−10.75	0.015^a^
		Total energy dissipation (J/kg)	−0.56 ± 0.11	−0.43 ± 0.15	−0.13	0.008^a^
lower extremity	Ankle joint	Mean energy dissipation (J/kg)	−0.87 ± 0.19	−0.73 ± 0.22	−0.14	0.211
		Contribution to total Energy dissipation (%)	26.22 ± 4.43	26.37 ± 4.71	0.15	0.948
	Knee joint	Mean energy dissipation (J/kg)	−1.61 ± 0.28	−1.36 ± 0.47	−0.25	0.093
		Contribution to total energy dissipation (%)	48.67 ± 4.06	49.58 ± 8.50	0.91	0.743
	Hip joint	Mean energy dissipation (J/kg)	−0.83 ± 0.18	−0.65 ± 0.20	−0.18	0.025^a^
		Contribution to total energy dissipation (%)	25.11 ± 3.90	24.05 ± 5.86	−1.06	0.678
		Total energy dissipation (J/kg)	−3.31 ± 0.47	−2.74 ± 0.55	−0.57	0.017^a^

Note: J/kg: Joules per kilogram. Difference value: The absolute value of the bionic shoe minus the absolute value of the normal shoe.

a means significance with *p* < 0.05.

**FIGURE 3:**
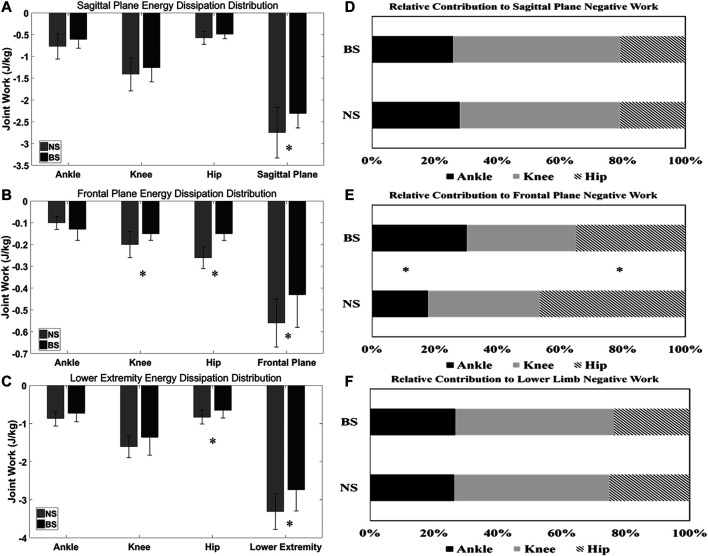
Comparison of mean energy dissipation and contribution to total energy dissipation in the sagittal plane, frontal plane, and lower extremity between NS and BS during single-landing phase. * refer to the significant difference between NS and BS (*p* < 0.05).

For the sagittal plane mean energy dissipation, Figure 3A shows that there are no differences in the ankle knee and hip joint, but BS depicted a significantly smaller sagittal plane total energy dissipation (*p* = 0.028) than NS during the landing phase.

For the frontal plane mean energy dissipation, Figure 3B shows that BS depicted a significantly smaller knee mean energy dissipation (*p* = 0.044), a significantly smaller hip mean energy dissipation (*p* = 0.000), a significantly smaller frontal plane total energy dissipation (*p* = 0.008) than NS during the landing phase.

For the lower limb mean energy dissipation, Figure 3C shows that BS depicted a significantly smaller hip mean energy dissipation (*p* = 0.025), a significantly smaller lower limb total energy dissipation (*p* = 0.017) than NS during the landing phase. There were no differences in the ankle and knee joint mean energy dissipation.

There were no differences in the relative contribution to total energy dissipation in the sagittal plane (Figure 3D) and lower limb (Figure 3F) negative work. For the relative contribution to frontal plane negative work, Figure 3E shows that BS depicted a significantly smaller relative ankle contribution (*p* = 0.006), a significantly smaller relative hip contribution (*p* = 0.015) than NS during the landing phase.

### Joint Reaction Force and Anterior Tibia Shear Force

**FIGURE 4:**
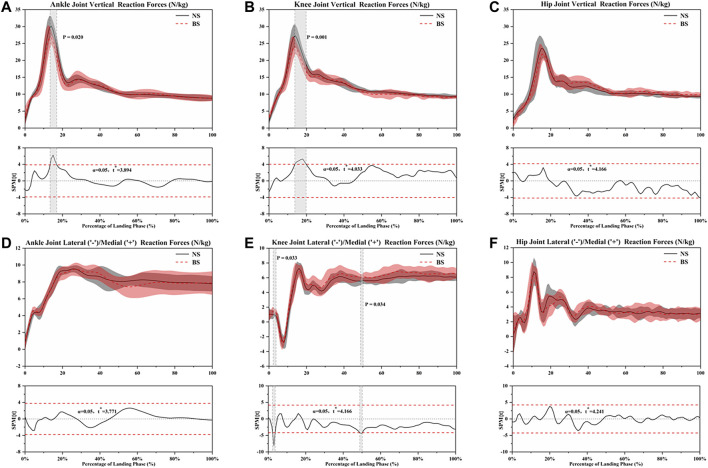
The Statistical Parametric Mapping (SPM) results between NS and BS during single-leg landing task, depicting the mean joint vertical reaction forces, mean joint lateral, and medial reaction force. Grey shaded areas indicate that there are significant differences (*p* < 0.05) between NS and BS during the landing phase.

For the ankle joint reaction force results, SPM analysis revealed that NS depicted a significantly greater vertical reaction force (Figure 4A) than BS during the 13.4–16.9% landing phase (*p* = 0.02). There were no differences in lateral and medial reaction force (Figure 4D). Table 4 shows that NS depicted greater peak vertical reaction forces (*p* = 0.001) than BS.

**TABLE 4:** Comparison of joint vertical reaction forces, joint medial reaction forces and anterior tibia shear force (means and standard) between NS and BS during single leg landing phase.

	Biomechanical variables	Normal shoe	Bionic shoe	Difference value	P
Ankle joint	Peak vertical reaction forces (N/kg)	30.39 ± 4.37	27.19 ± 3.58	−3.20	0.001^a^
	Peak medial reaction forces (N/kg)	9.61 ± 2.22	9.50 ± 2.38	−0.11	0.701
Knee joint	Peak vertical reaction forces (N/kg)	27.43 ± 4.75	24.13 ± 3.49	−3.30	0.004^a^
	Peak medial reaction forces (N/kg)	7.42 ± 2.28	7.34 ± 1.58	−0.08	0.693
Hip joint	Peak vertical reaction forces (N/kg)	24.34 ± 3.43	22.21 ± 4.46	−2.13	0.022^a^
	Peak medial reaction forces (N/kg)	8.74 ± 1.93	8.82 ± 1.61	0.08	0.998
Peak anterior tibia shear force (BW)		1.81 ± 0.27	1.62 ± 0.18	−0.19	0.001^a^

Note: N/kg: Newtons per kilogram; BW: Body Weight. Difference value: The absolute value of the bionic shoe minus the absolute value of the normal shoe.

a means significance with *p* < 0.05.

For the results of the knee joint reaction force, SPM analysis revealed that NS depicted a significantly greater vertical reaction force (Figure 4B) than BS during the 13.8–19.8% landing phase (*p* = 0.01), but there were no differences in lateral and medial reaction force (Figure 4E). Table 4 shows that NS depicted greater peak vertical reaction forces (*p* = 0.004) than BS.

For the hip joint reaction force results, SPM analysis revealed no differences in vertical reaction forces (Figure 4C), lateral and medial reaction force (Figure 4F) between NS and BS. Table 4 shows that NS depicted greater peak vertical reaction forces (*p* = 0.022) than BS.

**FIGURE 5:**
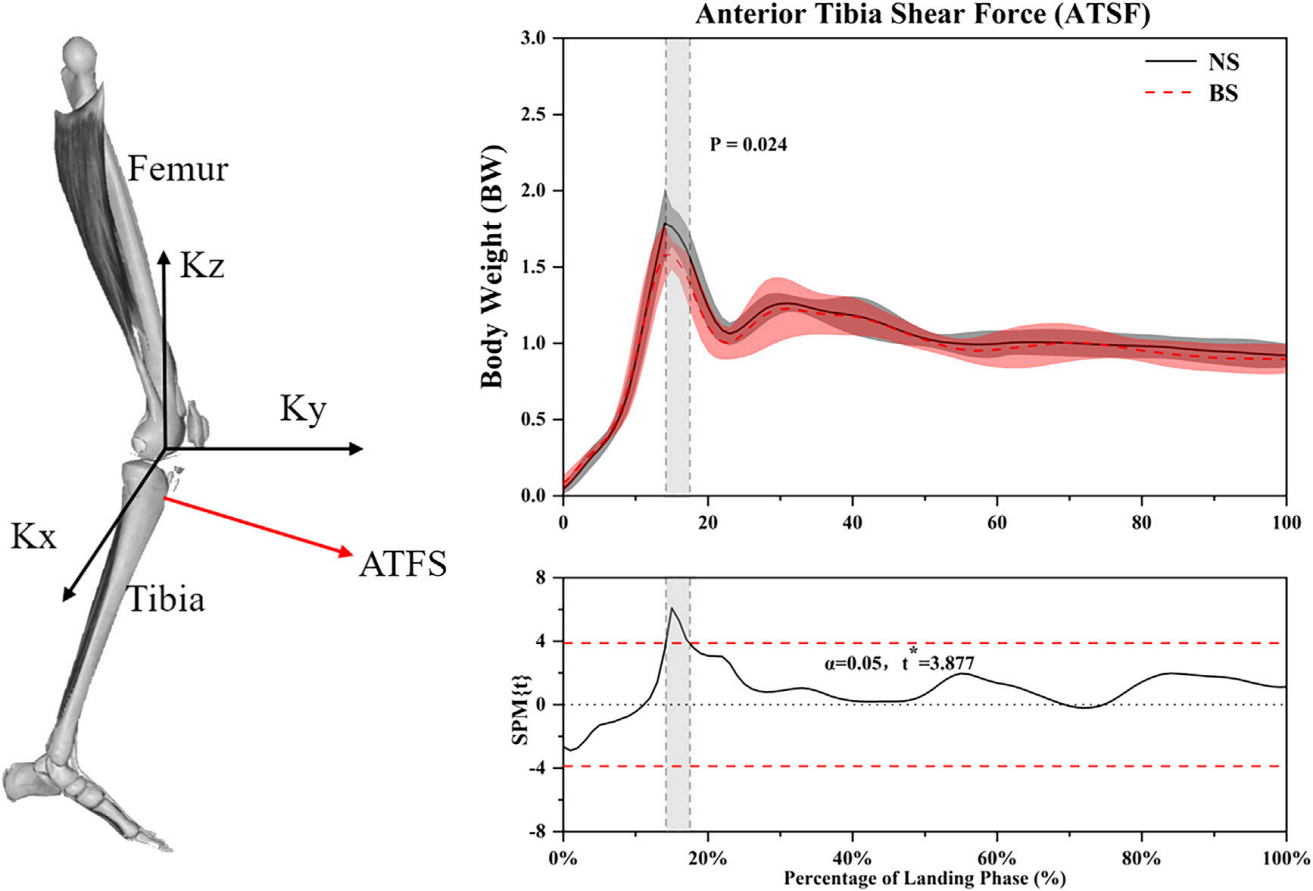
The Statistical Parametric Mapping (SPM) results between NS and BS during single-leg landing task, depicting the mean anterior tibia shear force. Grey shaded areas indicate that there are significant differences (*p* < 0.05) between NS and BS during the landing phase.

For the anterior tibia shear force results, SPM analysis revealed that NS depicted a significantly greater anterior tibia shear force (Figure 5) than BS during the 14.2–17.5% landing phase (*p* = 0.024). Table 4 shows that NS depicted greater peak anterior tibia shear force (*p* = 0.001) than BS.

## Discussion

This study aimed to investigate the effects of using BS on the biomechanics of the lower limb sagittal plane and frontal plane during a single-leg landing task. We hypothesized that the lower limb biomechanics with NS and BS might be different, and these differences may reduce landing injuries from the perspective of biomechanical analysis. Our results agree in part with the proposed hypothesis. The present study shows that BS changes the lower limb joint (ankle, knee hip) angle, joint energy work, and joint reaction forces compared to the NS during a landing task.

Unstable shoes are thought to absorb shock during initial contact, adjust postural control systems, and improve performance (Nigg et al., 2006b; Taniguchi et al., 2012; Sousa et al., 2013). Our study shows that the effects of bionic sole construction on lower limb kinematics exist in the greater initial contact knee flexion angle, the initial contact hip flexion angle, and peak hip flexion angle. Compared to other planes, the sagittal plane of motion amplitude was the largest, and the impact on lower limb injury was also the largest. Previous studies have shown that larger flexion angles of the knee and hip during landing may increase shock absorption and reduce joint reaction force, thereby reducing the risk of lower limb injury (Markolf et al., 1995; Lee et al., 2018; Xu et al., 2021a). In our study, BS depicted greater ankle, knee, and hip vertical reaction forces than NS, and reduced the risk of lower limb joint injuries. This subconscious increase in the flexion angle of joints could lead to dynamic changes, potentially preventing landing injuries. However, BS had no effect on the lower limb joints’ range of motion, which means BS mainly changed the knee and hip flexion angle throughout the landing phase.

For the BS showed a greater initial contact and peak knee and hip flexion angle during landing, we assume that there are several possible reasons for this. First of all, BS may generate a potential protective adaptation mechanism to reduce landing injury. The foot shape outsole of BS provides an unstable surface to simulate the instability of the barefoot to increase instability. The subjects had a certain understanding of the unstable response with BS landing after 2 weeks of adaptation in BS. The subject realized that BS would produce instability compared to NS when they performed the landing experiment. So they will consciously lower their body’s center of gravity during landing by increasing the initial contact flexion angle of the knee and hip to counteract this kind of instability. At the same time, the balance was maintained by consciously increasing the knee and hip flexion angle at the end of the landing phase (e.g., show a greater peak knee and hip flexion angle). We hypothesize that this conscious response (increasing the knee and hip flexion angle) acts as a protective mechanism to reduce landing injury. Therefore, it is worth further exploring whether the subjects can form a firm conscious memory through the intervention with BS for a long time so that even landing with NS can also stimulate this protection mechanism by increasing the flexion angle of the knee and hip joint.

We hypothesize that another possible cause is the pre-activation of some involuntary muscles. Muscle pre-activation is a mechanism whereby certain muscles are activated earlier than others for certain movements, and it can also be understood that muscles are activated before the movements occur (the normal condition is that the muscles are activated after the movements have occurred) (Júnior et al., 2010; Müller et al., 2010). Previous studies have shown that the pre-activation mechanism can reduce musculoskeletal injury to some extent by activating muscle activity in advance (Nigg et al., 2006a; Hashemi et al., 2010; Müller et al., 2010). Hashemi et al. found that ACL injury could be reduced during the landing phase of a jump by increasing the pre-activation of the quadriceps (Hashemi et al., 2010). Therefore, we assume that the pre-activation mechanism also existed in landing with BS. The body needs to use more muscles to maintain balance when it is unstable, and it does not need these voluntary muscles when it is stable (Nigg et al., 2012). These involuntary muscles (e.g., quadriceps and tibialis anterior) were activated when the subjects stood on the platform for the landing test with BS, thereby increasing the angle of initial contact with the knee and hip joints to reduce landing injury. However, it is only a speculative hypothesis, further studies are needed to prove whether the pre-activation mechanism applies to landing with BS.

In our study, subjects were asked to step off from the same height platform (35 cm), which generates the same potential energy. These potential energies were absorbed primarily by the muscular and skeletal systems as buffers. Compared to NS, BS increased muscle activity in the lower limbs, especially in the calf muscles, increasing the absorption of energy by the muscles (Devita and Skelly, 1992; Turbanski et al., 2011). The joints’ amount of work represents how much impact load is absorbed by the lower limbs’ skeletal system (Zhang et al., 2000). For the same potential energy, if the muscular system absorbs more, there will be less impact on the skeletal system. In our study, the results show that BS depicted a significantly smaller sagittal plane total energy dissipation and lower limb total energy dissipation than NS during the landing phase. It is worth noting that the energy transfer and impact load can only be absorbed by the lower limb ends in contact with the ground when landing with a single leg, which is undeniable that single-leg landing carries a significant risk of injury. Therefore, the energy dissipation mechanism of BS in single-leg landings also has good damage prevention performance. At the same time, the BS depicted a significantly smaller knee and hip mean energy dissipation than NS in the frontal plane. A possible reason for this may be that the subject compensates by coordinating the muscular system to counteract the muscular disturbance mechanism of instability using BS. Our study suggests that BS may increase the muscle absorption impact load and reduce the impact of energy load on the bone joints of the lower limbs’ bone joints to reduce the risk of various bone joints and ligament injury.

The foot shape outsole of BS creates an unstable environment to increase movement sensation (Lohrer et al., 2008; Zhou et al., 2018). Our results also demonstrate the role of this kind of movement sensation in terms of altering landing strategies. Another interesting result is that SPM analysis revealed that NS depicted a significantly greater vertical reaction force and anterior tibia shear force than BS during the 13.8–19.8% and 14.2–17.5% landing phase, respectively. Considering that current kinematics and kinetics analysis tend to focus on the peak data and assume that the peak data moment is consistent with the moment when the movement risk is generated, the importance of other continuous data moments is often ignored. Therefore, one-dimensional statistical parameter mapping (SPM) analysis is used to achieve the purpose of time series analysis of continuous data over a landing phase (Pataky et al., 2013). Previous studies have shown that ACL injury happens 30–50 milliseconds (about 15–25% landing phase) after initial ground contact (Krosshaug et al., 2007; Hogg et al., 2020; Xu et al., 2021b). The anterior tibia shear force acting on the proximal tibia is considered to be the primary source of ACL load, which is also the main shear force that causes ACL injury (Shelburne et al., 2004). When these differences occur almost within the time frame of ACL injury (30–50 milliseconds after landing), BS may be consistent with the trend associated with a lower risk of ACL injury. This also seems to be related to the increased knee and hip flexion angles, and the kinematics caused by BS leads to a series of kinetic changes. Therefore, the changes of knee joint vertical reaction force and the anterior tibia shear suggest that the uniqueness of BS’s design may serve as the shoe application developed to prevent ACL injuries.

Therefore, the foot shape outsole of BS can change the biomechanics of lower limbs during landing tasks, thereby reducing the risk of lower limb injury, including some skeletal muscle injuries. Previous studies have shown that bionic shoe structures contribute to specific training and rehabilitation programs because of postural control availability in the human body and the ability to readjust the lower limb biomechanics (Devita and Skelly, 1992; Gu et al., 2014). Simultaneously, the role of BS is also reflected in its various internal and external interference, autonomous and involuntary control (Jiang et al., 2021). Our study has shown that BS can adjust the lower limb kinematics at initial contact and then readjust the joint work and joint reaction force landing strategies. Therefore, BS with unstable shoe structures may have great development and application value, which may help athletes reduce lower limb injury risk. These changes in the landing mechanism may be due to the activation of some subjects’ involuntary muscles when they single-leg land on the BS during a period of adaptation, but more detailed reasons need to be further investigated. Our current research on bionic shoes has not reached potential levels of overall application, so we need to further explore the internal mechanism of bionic shoes. For instance, different sports have different needs for shoes, which require multiple considerations and designs in combination with other sporting contexts. Therefore, we should make efforts to improve bionic shoes based and expand existing theories for future developments. These initiatives when achieved, will truly transform bionic shoes into exciting products with a better functional concept and structural design.

Our present study has some limitations that should be considered. First of all, the subjects in our experiment adapted to the BS in the short term (1 h per day for 2 weeks), and they lacked long-term adaptation. Compared with short-term adaptation, long-term adaptation may enable subjects to adapt to bionic shoes more comprehensively, thus showing different biomechanical characteristics. Future research should investigate adaptation time to explore the underlying biomechanical change mechanism more deeply and completely. We determined the participant’s familiarity before testing through the subject’s self-description, but no related self-reported data (such as the extent of comfort and consistency of performance) has been recorded. Further studies should consider recording these data to better quantify the effect. Our subjects were healthy males, and the study did not include female subjects. Previous studies have shown that females exhibit different landing strategies when landing from heights, which is mainly reflected in poor postural stability and a greater risk of lower limb injury (Decker et al., 2003). Therefore, we need to consider female subjects in future studies. Furthermore, given the force traces shown, it would be useful to support the ACL injury argument with time-related variables, such as time to stabilization and loading rate. These variables should also be considered in future studies. At present, the possible reasons for the BS showed a greater initial contact and peak knee and hip flexion angle during landing is only a speculative hypothesis, the specific mechanism still needs to be explored by other approaches. For instance, electromyography testing is used to track the difference in muscle activity of the lower extremities between BS and NS over a long period to prove muscle pre-activation mechanism. Finally, the sole’s hardness greatly influences the lower limbs’ biomechanics, but we selected only one kind of hardness of the sole for our experimental shoe. Therefore, sole materials of different hardness should also be taken into consideration during future research.

## Conclusion

In summary, our study investigated the effects of BS on the biomechanics of the lower limb sagittal plane and frontal plane during a single-leg landing task. We found that BS depicted a significantly greater knee and hip flexion angle, a significantly smaller sagittal plane, frontal plane, and lower limb mean energy dissipation, a significantly smaller ankle and knee vertical reaction force, a significantly smaller anterior tibia shear force than NS during the landing phase. The foot shape outsole of BS can change the lower limb kinematics at the initial contact and then readjust the landing strategies in joint work and joint reaction force, thereby reducing the risk of lower limb skeletal muscle injury. Therefore, BS has great development and application potential, which can help athletes during training and competition and reduce lower limb injury risk. Future research should also consider adaptation time, female subjects, and the sole’s hardness to explore the underlying biomechanical change mechanisms more comprehensively.

## Data Availability

The datasets generated for this study are available on request to the corresponding author.
